# Linking attentional processes and conceptual problem solving: visual cues facilitate the automaticity of extracting relevant information from diagrams

**DOI:** 10.3389/fpsyg.2014.01094

**Published:** 2014-09-29

**Authors:** Amy Rouinfar, Elise Agra, Adam M. Larson, N. Sanjay Rebello, Lester C. Loschky

**Affiliations:** ^1^Department of Physics, Kansas State UniversityManhattan, KS, USA; ^2^Department of Psychology, University of FindlayFindlay, OH, USA; ^3^Department of Psychological Sciences, Kansas State UniversityManhattan, KS, USA

**Keywords:** overt visual attention, physics education, problem solving, visual cognition, automaticity

## Abstract

This study investigated links between visual attention processes and conceptual problem solving. This was done by overlaying visual cues on conceptual physics problem diagrams to direct participants’ attention to relevant areas to facilitate problem solving. Participants (*N* = 80) individually worked through four problem sets, each containing a diagram, while their eye movements were recorded. Each diagram contained regions that were relevant to solving the problem correctly and separate regions related to common incorrect responses. Problem sets contained an initial problem, six isomorphic training problems, and a transfer problem. The cued condition saw visual cues overlaid on the training problems. Participants’ verbal responses were used to determine their accuracy. This study produced two major findings. First, short duration visual cues which draw attention to solution-relevant information and aid in the organizing and integrating of it, facilitate both immediate problem solving and generalization of that ability to new problems. Thus, visual cues can facilitate re-representing a problem and overcoming impasse, enabling a correct solution. Importantly, these cueing effects on problem solving did not involve the solvers’ attention necessarily embodying the solution to the problem, but were instead caused by solvers attending to and integrating relevant information in the problems into a solution path. Second, this study demonstrates that when such cues are used across multiple problems, solvers can automatize the extraction of problem-relevant information extraction. These results suggest that low-level attentional selection processes provide a necessary gateway for relevant information to be used in problem solving, but are generally not sufficient for correct problem solving. Instead, factors that lead a solver to an impasse and to organize and integrate problem information also greatly facilitate arriving at correct solutions.

## INTRODUCTION

This study investigated links between visual attention processes and conceptual problem solving. This is challenging, because most of what we know about attention has to do with its lower-level perceptual processes, and most of what we know about problem solving has to do with much higher-level cognitive processes. Thus, forging a link between lower-level perception and higher-level cognition is difficult. A vast literature has developed over the past 40 years explaining the low-level stimulus factors that capture attention and eye movements, and the effects this has on early visual perceptual processes. For example, motion has been shown to reliably capture eye movements (overt attention; Carmi and Itti, 2006; Mital et al., 2010), as mediated by the superior colliculus in primates (Kustov and Robinson, 1996; Findlay and Walker, 1999; Boehnke and Munoz, 2008), and the optic tectum in lower animals, including toads (Borchers and Ewert, 1979). In turn, selective attention has been shown to improve perceived brightness, acuity, and contrast sensitivity (Carrasco et al., 2000, 2002; Cameron et al., 2002), as mediated by an increased signal-to-noise ratio of cells as early as the primary visual cortex (Fischer and Whitney, 2009; Pestilli et al., 2011). However, despite the tremendous strides that have been made in understanding the low-level causes and effects of visual selective attention, much less is known about high-level cognitive causes and effects of visual selective attention. Admittedly, a sizeable body of research has shown strong relationships between tasks and selective attention, as measured by eye movements (Foulsham and Underwood, 2007; Henderson et al., 2007; Einhäuser et al., 2008), and between selective attention, as measured by eye movements, and memory (Hollingworth and Henderson, 2002; Zelinsky and Loschky, 2005; Pertzov et al., 2009). Nevertheless, far less research has investigated such causal relationships between visual selective attention and eye movements on the one hand, and quintessentially higher-level cognitive processes such as those involved in problem solving, on the other.

In the current study, we specifically investigate the relationships between visual selective attention and the cognitive processes involved in solving physics problems, which are among the most intellectually and cognitively demanding that human beings are capable of engaging in. Indeed, one might reasonably ask whether such low-level perceptual functions as those involved in selective attention could really play much of a role in such a high-level cognitive task. However, several studies over the last decade have shown exactly that, namely that cueing people’s attention in specific ways while they solve insight problems can significantly affect their solution accuracy (Grant and Spivey, 2003; Thomas and Lleras, 2007, 2009). In the current study, we have investigated these processes in the context of learning from problem solving. However, evidence of learning, as shown by increased performance on problem solving tasks alone, while clearly implicating memory formation, cannot elucidate the links between online attentional selection and the higher-level cognitive processes involved in physics problem solving. We therefore elucidated the online processes that link attention selection and physics problem solving by using eye movement data in conjunction with increases in problem solving performance.

## BACKGROUND

### SELECTIVE ATTENTION AND EYE MOVEMENTS

We assume that eye movements are linked to attentional selection as proposed by the rubber band model of eye movements and attention (Henderson, 1992, 1993). Specifically, at the beginning of each eye fixation, attention is aligned with the point of fixation (van Diepen and d’Ydewalle, 2003; Glaholt et al., 2012; Larson et al., 2014), but by roughly 80 ms before the next eye movement, covert attention is shifted to the to-be-fixated object (Kowler et al., 1995; Deubel and Schneider, 1996; Caspi et al., 2004), after which the eyes make a saccade to the newly attended object. Thus, although attention may be at a different location than the point of fixation (especially in the last 80 ms of a fixation, called covert attention; Caspi et al., 2004), if the eyes are sent to a location, we know that attention was there at the beginning of the fixation. One can therefore retrospectively measure the location of attentional selection by measuring eye fixation locations, called overt attention.

As noted earlier, research on attentional selection has made tremendous strides in explaining the effects of stimulus characteristics, or bottom-up influences, on overt attention. These studies have shown that stimulus saliency, as measured by contrast along various feature dimensions coded by early visual cortex (e.g., luminance, color, orientation, and motion), plays a moderately strong causal role in determining where the eyes are sent (Irwin et al., 2000; Itti and Koch, 2000; Mital et al., 2010). Other research has shown non-stimulus-based effects, or top-down influences, on overt attention. These top-down influences can be further divided between those that are involuntary and automatic, based on experience and learning, called mandatory top-down processes, and those that are voluntary and effortful, called volitional top-down processes (Baluch and Itti, 2011). Numerous studies have shown evidence of mandatory top-down effects on overt attentional selection in scenes (e.g., attention to stop signs when they are in expected locations, such as intersections, but not in unexpected locations, such as the middle of a block; Theeuwes and Godthelp, 1995; Shinoda et al., 2001). A separate body of research has shown effects of volitional top-down processes on overt attention in more laboratory-based tasks (e.g., the anti-saccade task, in which one looks in the opposite direction from a salient visual stimulus; Everling and Fischer, 1998). Overall, mandatory top-down processes have been shown to generally have a stronger influence on overt attentional selection than bottom-up visual saliency (Foulsham and Underwood, 2007; Henderson et al., 2007; Einhäuser et al., 2008). Conversely, because volitional top-down processes require executive attentional and working memory (WM) resources, they generally have weaker effects on overt attentional selection than bottom-up saliency, as shown by the antisaccade task, in which the sudden appearance of a simple stimulus is very difficult to avoid reflexively looking at, while it takes a conscious effort to looking in the opposite direction (Guitton et al., 1985; Mitchell et al., 2002). Nevertheless, a far fewer number of studies have investigated the relationships between bottom-up and top-down processes and overt attentional selection in higher-level cognitive tasks such as problem solving.

### COGNITIVE PROCESSES INVOLVED IN PROBLEM SOLVING

In order to discuss the relationship between overt attentional selection and the cognitive processes involved in problem solving, we must first specify what those higher-level cognitive processes might be. We are particularly interested in the cognitive processes involved in conceptual problems requiring insight, in which the solution is not immediately apparent, and solvers cannot simply adopt an algorithmic approach to finding a solution (Duncker, 1945; Ohlsson, 1992; Jones, 2003). Ohlsson’s (1992) representational change theory provides a framework to understand the cognitive mechanisms involved in solving problems that require conceptual insight, rather than purely algorithmic computation. This framework lends itself to our work on conceptual problem solving. Specifically, the problems we study are conceptual in nature because they require the solvers to recognize the appropriate physics concepts to apply. Recognition of the appropriate concept often comes to the solver in a moment of insight. While encoding the problem, the solver activates (apparently) relevant prior knowledge, which is used to construct a mental representation of the problem. This representation is then used to find a path to the solution. However, in insight problems, solvers commonly make several unsuccessful attempts to solve the problem, which forces them into an impasse, in which they realize that no path to the solution is apparent. In order to break the impasse, the solver must often restructure their mental representation of the problem in order to find a viable solution path. This produces the insight that then rapidly leads to solving the problem. Ohlsson’s (1992) theory provides a good framework for understanding a number of important cognitive processes involved in insight problem solving, but is relatively silent with regard to what roles, if any, attentional selection plays in problem solving.

### PRIOR RESEARCH ON OVERT ATTENTIONAL SELECTION AND PROBLEM SOLVING

Prior research on eye movements and problem solving has shown that overt attention can illuminate the cognitive processes involved in problem solving (Epelboim and Suppes, 2001; Knoblich et al., 2001, 2005; Grant and Spivey, 2003; Jones, 2003; Thomas and Lleras, 2007, 2009; Bilalić et al., 2008; Eivazi and Bednarik, 2010, 2011; Madsen et al., 2012, 2013a,b; Lin and Lin, 2014; Susac et al., 2014). However, we are particularly interested in two directions of causal relationships between overt attentional selection and the higher-level cognitive processes involved in problem solving: (1) the causal relationship starting from higher-level cognitive processes involved in problem solving and ending with attentional selection; and (2) the reverse causal relationship starting from attentional selection and ending with the higher-level cognitive processes involved in problem solving. A relatively small number of studies have investigated each of these relationships, with some speaking more to the effect of higher-level cognitive processes in problem solving on attentional selection (Epelboim and Suppes, 2001; Knoblich et al., 2001; Madsen et al., 2012), and others speaking more to the effect of attentional selection on higher-level cognitive processes in problem solving (Epelboim and Suppes, 2001; Cameron et al., 2002; Grant and Spivey, 2003; Tai et al., 2006; Thomas and Lleras, 2007, 2009; Lin and Lin, 2014; Susac et al., 2014).

Research on the effect of the cognitive processes involved in problem solving on overt attentional selection has shown that mandatory top-down processes based on prior knowledge can enable solvers to rapidly attend to relevant information when solving a problem (Epelboim and Suppes, 2001; Madsen et al., 2012). In the most extreme cases, based on prior knowledge, an expert may attend to the relevant information in a problem within the time frame of a single eye fixation, while a novice may instead take much more time while attending to various sources of irrelevant information (Charness et al., 2001; Reingold et al., 2001). Just as importantly, however, even if the solver has previously activated irrelevant knowledge, leading to an impasse, restructuring the problem representation can lead to shifting overt attention away from irrelevant information to relevant but previously ignored information (Knoblich et al., 2001; Jones, 2003).

Research on the effect of attentional selection on the cognitive processes involved in problem solving suggests that there are at least two qualitatively different types of effects. First, attentional selection can lead either to processing relevant information, which facilitates problem solving by activating relevant domain knowledge, leading to finding a viable solution path, or processing irrelevant information, which impedes problem solving by activating irrelevant knowledge, leading to an incorrect solution path (Grant and Spivey, 2003; Thomas and Lleras, 2007, 2009; Madsen et al., 2012, 2013a). This effect of attentional selection on problem solving determines whether or not the solver, in a manner of speaking, gets through the starting gate to finding a viable solution path. Second, if a solver has gotten through the starting gate by attending to relevant information, further attentional selection of aspects of that relevant information appears to be important for not only extracting further relevant information, but also refreshing their WM representations used in finding the solution path. Here, we assume that problem solving occurs in WM (Ohlsson, 1992; Epelboim and Suppes, 2001), and that WM has a limited capacity (Baddeley, 1994; Luck and Vogel, 1997; Cowan, 2001). Thus, if the process of finding a viable solution path involves establishing relationships between numerous conceptual entities, solvers may experience difficulties caused by exceeding their WM capacity (Epelboim and Suppes, 2001). Because maintaining representations in WM requires attention (Cowan, 2001), one can refresh WM representations by attending to them (Hale et al., 1996; Awh et al., 1998; D’Esposito et al., 1999), for example by repeatedly refixating the eyes on the to-be-processed items (Zelinsky et al., 2011). Thus, during problem solving, attentional selection, as evidenced by refixating relevant information, can facilitate finding a solution path by refreshing the WM representations for the fixated items (Epelboim and Suppes, 2001; Tai et al., 2006; Lin and Lin, 2014; Susac et al., 2014).

A different way in which overt attentional selection can facilitate problem-solving processes in WM is through sustained attention, which involves inhibiting overt and covert attentional shifts. Specifically, when a solver is engaged in complex problem solving processes in WM, longer than normal processing times are sometimes needed in order to attend to the current contents of WM. In those cases, it would be counter-productive to move attention and the eyes to a new location, which automatically triggers extracting the new information there into WM (Belopolsky et al., 2008), potentially displacing some of the current WM contents (Zelinsky and Loschky, 2005). Instead, the solver may inhibit moving the eyes, resulting in a longer eye fixation at the current location (Findlay and Walker, 1999). Thus, during the process of breaking an impasse (i.e., the moment of insight), problem solvers will often produce longer fixation durations, rather than making more fixations on different items (Knoblich et al., 2001; Velichkovsky et al., 2002; Jones, 2003).

The above discussion sets the stage for discussing our previous work on overt attentional selection and physics problem solving. Our research was inspired by the groundbreaking work of Thomas and Lleras (2007, 2009), which demonstrated that shifting overt or covert attention in ways that embody the solution to Duncker’s (1945) tumor problem improved performance on it, even without solvers being aware of the relevance of the cueing to finding the problem’s solution. The concept of having attentional movement trajectories embody the solution to a problem, while powerful, may not apply to solving a wide array of problems. However, the simpler relationship between what is selected for visual attention and how that affects problem solving cognitive processes can be investigated in most if not all problems involving figures. Our particular approach to investigating this issue has been to use specific physics problems that contain two distinct regions, those associated with well-documented misconceptions and those associated with correctly solving the problems. In this way, a direct connection can potentially be found between overt attentional selection and problem solving cognitive processes. The results of these studies showed that when attempting to solve such problems, solvers’ overt attention was strongly guided by mandatory top-down processes (prior knowledge, either correct or mistaken) to either the relevant or irrelevant regions respectively (Madsen et al., 2012, 2013a). Importantly, those who overtly attended more to the relevant information were more likely to correctly solve the problems, and those who overtly attended to regions associated with well-documented misconceptions more frequently gave incorrect answers in line with those misconceptions. This raised the question of whether guiding solvers’ overt attention to the relevant information would facilitate their correctly solving those or similar problems. In one study, we modified the bottom-up visual saliency (as measured by a computational model) of the relevant vs. irrelevant regions in physics problems (by increasing or decreasing the luminance contrast of the lines in the problem diagrams; Madsen et al., 2013b). Interestingly, we found that solvers’ mandatory top-down processes (prior knowledge) guided their overt attention, overwhelming any potential effects of stimulus saliency (Madsen et al., 2013b). Nevertheless, as before, those who attended more to relevant information were more likely to correctly solve the problems (Madsen et al., 2012). In another study, we used highly salient visual cues (moving colored dots) that mimicked the overt attention shifts that correct solvers often made while solving those problems, and asked the solvers to follow the dots with their eyes (without explaining why) while they solved the problems (Madsen et al., 2013a). We found that the moving dot cues often guided solvers’ overt attention to the relevant areas (assumedly based on both bottom-up stimulus saliency and volitional top-down processes). However, the highest percentage of cued participants answering a training problem correctly was 41%, which was not significantly higher than the 32% in the uncued condition. Further, the cued participants significantly outperformed the uncued participants on the training problems in only one of the four problem sets. Likewise, the cued participants significantly outperformed the uncued participants on the transfer problem in only one of the four problem sets (Madsen et al., 2013a). Thus, getting solvers through the starting gate, by guiding their overt attention to relevant information, was often insufficient to facilitate correct problem solving.

In sum, our prior work has shown that higher-level cognitive processes involved in physics problem solving very strongly guide solvers’ overt attentional selection. Furthermore, overt attentional selection of relevant (rather than irrelevant) information is associated with a higher probability of correctly solving such problems. However, we have also shown that simply guiding solvers’ overt attention to relevant areas of physics problems is often insufficient to correctly solve those problems, or transfer problems similar to them.

## THE CURRENT STUDY

Our prior results described above left important open research questions. Specifically, although previous work clearly showed that higher cognitive processes strongly affect attentional selection during insight problem solving, much less clear is the degree to which attentional selection, as guided by visual cues, can strongly affect higher-level cognitive processes involved in conceptual physics problem solving.

We therefore considered our previous results in terms of their relationship to Ohlsson’s (1992) model of insight problem solving, which suggested that we make several changes to our methodology. These changes were done in order to facilitate both the guidance of overt attention to relevant information, and the use of that information to restructure solvers’ representations of the problems and find correct solution paths. Specifically, although several previous studies had shown that the solvers’ success rate in solving Dunker’s radiation problem could be increased by their cueing attention without explaining why (Grant and Spivey, 2003; Thomas and Lleras, 2007, 2009), we repeatedly found that simply guiding solvers’ attention to the relevant information in a problem was insufficient for them to arrive at a correct solution path (Madsen et al., 2012, 2013a). Thus, we decided to explicitly indicate to solvers that the cues were relevant to solving the problems, by referring to the cues as “hints,” which were meant to help them.

In addition, we previously observed that solvers who were incorrect on the first problem in a set of similar problems tended to repeatedly use the same incorrect solution path for every problem in the set. Thus, in terms of Ohlsson’s (1992) model of insight problem solving, the solvers were apparently not facing an impasse that would force them to restructure their faulty representation of the problem. This points out a difference between our problems and many common insight problems, for example Maier’s two-string problem (Maier, 1931). In our problems the solver may not know that they have failed to reach the goal state, whereas many insight problems are structured such that failure to reach the correct goal state is self-apparent. We therefore decided to provide the solvers with correctness feedback (i.e., saying “correct” or “incorrect” without explaining why) after they gave their answer to each problem. This would facilitate their entering an impasse for those problems they solved incorrectly, with the idea that solvers could then potentially break their impasse by restructuring their representations of those problems. In such cases, the visual cues could direct solvers’ attention to relevant information, which could activate previously dormant relevant knowledge from long-term memory, enabling the solver to create a new representation for the problem that could break the impasse. In order to determine the individual effects of correctness feedback and visual cueing on overt attention and problem solving, we manipulated both factors independently in our experimental design.

We also incorporated a key idea from de Koning et al’s. (2009) model of attentional cueing for learning, specifically that cues can be used not only to facilitate selecting important information for attention, but also to facilitate integrating information across different regions within a problem. For instance, cues can facilitate making comparisons between different elements of a problem, such as comparing the distance traveled at different points in time, or comparing the slopes of two curves on a graph. Such cues still function to direct the solvers’ attention, but go beyond simply directing attention to a location in space by symbolically indicating the types of information to attend to at those locations, and between different locations over time.

In order to measure changes in attentional selection and problem solving over time (i.e., learning), as in our previous studies (Madsen et al., 2013a), for each base problem, we created a series of similar problems, which will be discussed in the Methods section. Furthermore, as in our previous studies (Madsen et al., 2013a), in order to test for more than just superficial learning, we created transfer problems that used the same underlying reasoning (and solution paths), but had somewhat different surfaces features. In addition, we did not use cues on either the initial problem for each sequence, or on the transfer problem for that sequence, in order to measure both overt attentional selection and problem solving cognitive processes in the absence of cueing.

Given the above discussion, it is worth considering what changes in perceptual and higher level cognitive processing might occur as a consequence of learning engendered by cueing problem solvers on successive trials, each with a similar problem that differs only minimally in its surface features from the previous problem, and then testing on a transfer problem that differs more substantially in its surface features. Changes in solvers’ problem representations could be measured off-line in terms of giving correct answers on the transfer problems by solvers who had given incorrect answers on the initial problem for that problem type. Of particular interest for the current study, we can also measure such changes in the solvers’ problem representations on-line in terms of eye movement data, for example by solvers overtly attending to relevant information on transfer problems that they had previously ignored in the initial problem of that problem type. A more specific hypothesis is that solvers who had previously been cued would have learned to attend to the relevant information, and thus spend more time processing the relevant information on the transfer problem than those solvers who had not been previously cued. We will call this the processing priority hypothesis. Interestingly, however, an alternative competing hypothesis is suggested by considering a further aspect of learning, namely automatization (Schneider and Shiffrin, 1977), which could be measured in terms of increased efficiency of information extraction and integration into a solution path in WM. Assumedly, repeatedly attending to relevant information and using it to create a similar correct solution path would engender greater automaticity (i.e., efficiency) in performing each of these perceptual and cognitive processes. Automatization as shown by eye movements could be measured in terms of fixation durations, which are generally taken as an indication of processing difficulty (Rayner, 1998; Nuthmann et al., 2010). Thus, to the degree that relevant information extraction and integration is automatized, it should produce shorter fixation durations. More specifically, an alternative hypothesis is that solvers who had previously been repeatedly cued should process the relevant information in a more automatized manner, and thus have shorter fixation durations on the relevant information on the transfer problem than those solvers who had not been previously cued. We will call this the automatization hypothesis.

## MATERIALS AND METHODS

### PARTICIPANTS

The participants in this study (*N* = 80, 44 males, 36 females) were enrolled in a first semester algebra-based physics course and were compensated with course credit. All participants had uncorrected or corrected-to-normal vision.

### STIMULI

**FIGURE 1:**
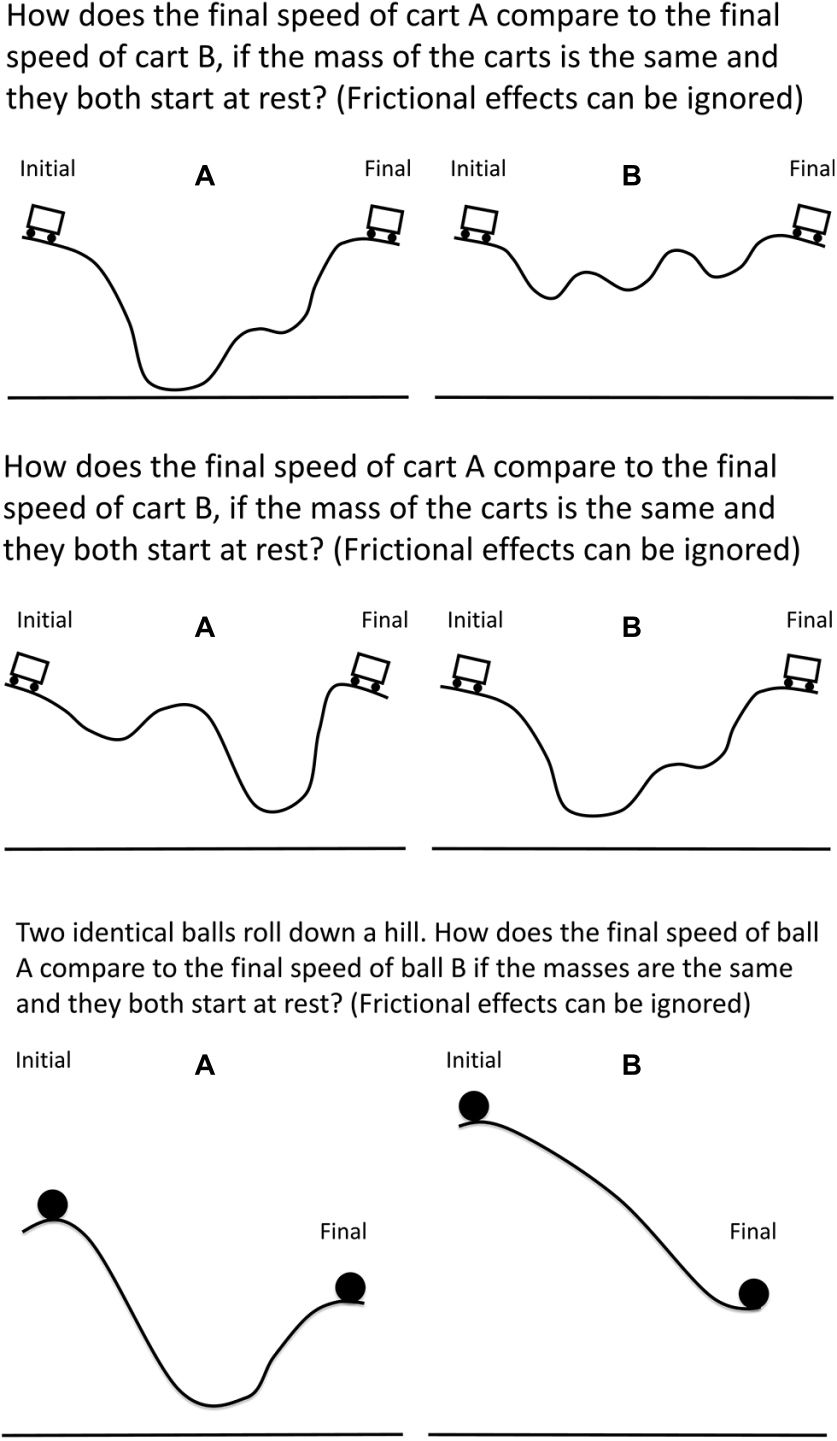
**Example of an initial problem (top), one of the six training problems (middle), and a transfer problem (bottom)**.

Four problem sets were investigated in this study and covered the topics of speed and energy conservation. Participants covered the requisite material in their course before being recruited to participate in the study. The problem sets examined in this study all contained diagrams with features consistent with novice-like answers documented in the literature and separate areas relevant to correctly solving the problem (Madsen et al., 2012). Each set consisted of eight problems: an initial problem, six isomorphic training problems, and a transfer problem. The transfer problem assessed the same concept as the other problems in the set, but had different surface features (e.g., Reed, 1993). An example of a problem set is provided in **Figure 1**.

**FIGURE 2:**
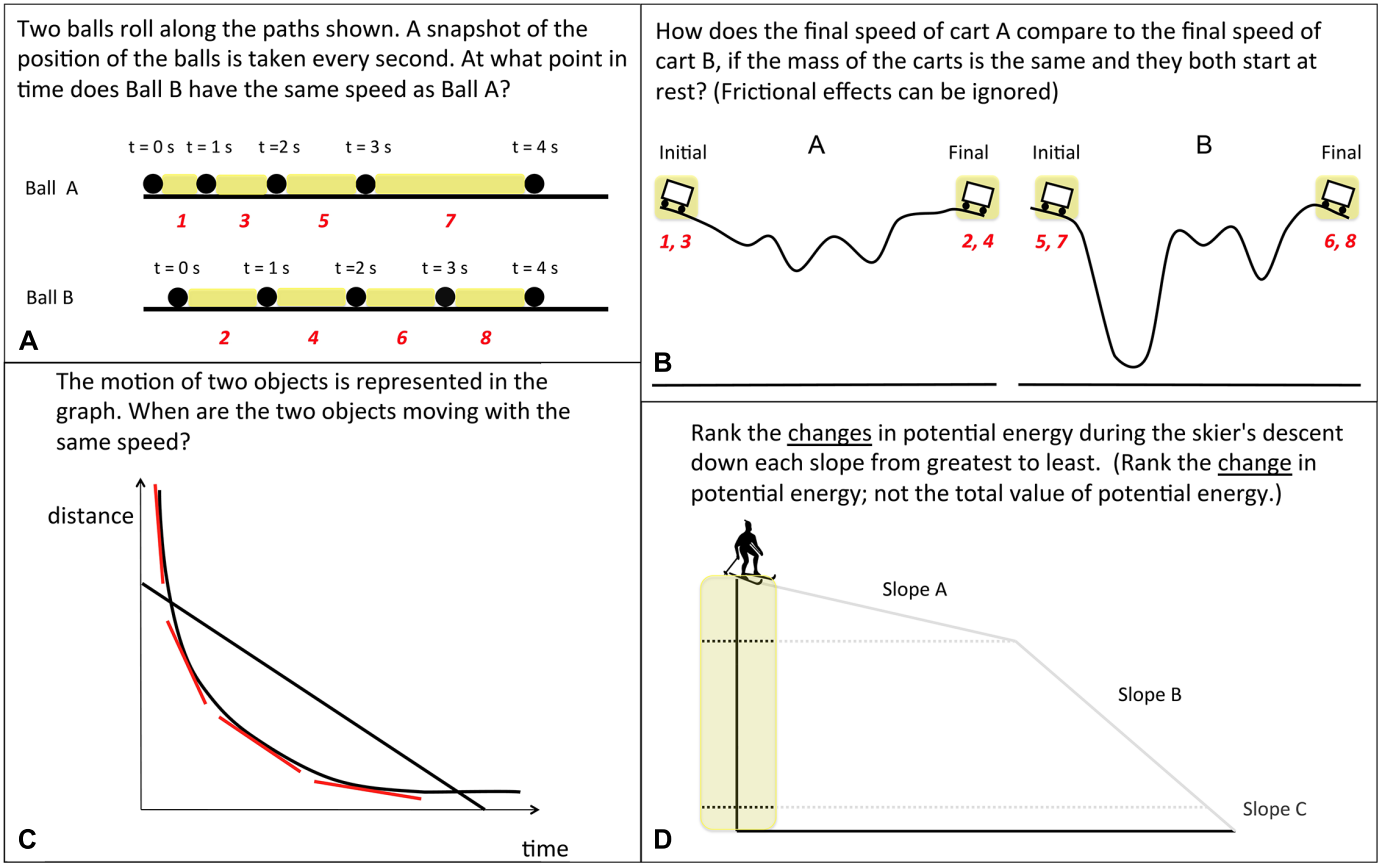
**Examples of training problems with the cue superimposed from the **(A)** ball, **(B)** cart, **(C)** graph, and **(D)** skier problem sets, respectively.** The cue is represented by the colored shapes (and grayed slopes for the Skier). The cues lasted for 8 s, and the numbers indicate the order in which the shapes appeared on the ball and cart problems. The critical information needed to solve each problem is as follows: in problem **(A)** the balls will have the same average speed during the time interval in which they travel the same distance. In problem **(B)** the shapes of the tracks are irrelevant to the carts’ final speeds due to the lack of friction, so it is only necessary to compare the starting and ending heights of the carts. In problem **(C)** the two objects represented in the graph will travel at the same speed when they have equal slopes, as speed is the rate of change of position. In problem **(D)** the change in potential energy depends only on the change in vertical height for each segment.

The cues were described to the participants as hints, which were meant to help them solve the problem. When ready to view the cue, the participants pressed a button. All participants in cued conditions were required to view the cue at least once, but there was no limit on the number of times they could replay it. Explanations of the critical information needed to solve each problem, along with examples of the cues for those problems are provided in **Figure 2**.

The problems were presented to participants on a computer screen. The screen had a resolution of 1024 × 768 pixels and a refresh rate of 85 Hz. The images subtended 33.3°× 25.5° of visual angle. Participants used a chin and forehead rest that was 24 inches from the screen. Eye movements were recorded with an EyeLink 1000 desktop mounted eye-tracking system which had an accuracy of less than 0.50° of visual angle.

### DESIGN AND PROCEDURE

This study was part of a larger study in which we investigated the effect on problem correctness due to both feedback and visual cues (Rouinfar et al., 2014). In this paper we focus on the analysis of the eye movement data, though we use accuracy data to show evidence of learning to make arguments linking the eye movements to learning.

Each participant took part in an individual session lasting 50–60 min. At the beginning of the session, participants were given a short explanation of the goal of the interview and given instructions. The eye tracker was calibrated to the individual using a nine-point calibration and validation procedure, with a threshold agreement of 0.5° visual angle required to begin the experiment.

Participants were randomly assigned either a cued condition (*N* = 38, 22 males, 16 females) or an uncued condition (*N* = 42, 22 males, 20 females). Those in the cued conditions saw colored shapes superimposed on the diagrams of the training problems for 8 s, but not on the transfer problems. All participants worked through four sets of problems. The order of the problem sets and the training problems within each set was randomized. Participants were told to spend as much time as they needed on each question and to give a verbal answer and explanation whenever they were ready. The participants were able to point to areas on the computer screen while explaining their answers if necessary. The experimenter used a pre-defined rubric to determine if the given answer and explanation were correct or incorrect. The experimenter would ask for clarification if the participant provided a vague answer or explanation. To be considered correct, the responses were required to contain both the correct answer and scientifically correct explanation.

## RESULTS

The current study reports on eye movement data collected in an experiment reported in more detail in Rouinfar et al. (2014). That experiment factorially manipulated both cueing and feedback and found significant main effects of both factors, but no interaction between them, on accuracy of physics problem solving. That study did not report on the eye movement data, which is the focus of the current study. The current study analyzed the effects of both cueing and feedback, but found no significant main effects of feedback, nor any interactions of feedback with cueing, on any eye movement measures. Therefore, to streamline our description of our results, we have collapsed across the feedback factor and will not discuss that factor further.

### CORRECTNESS

**FIGURE 3:**
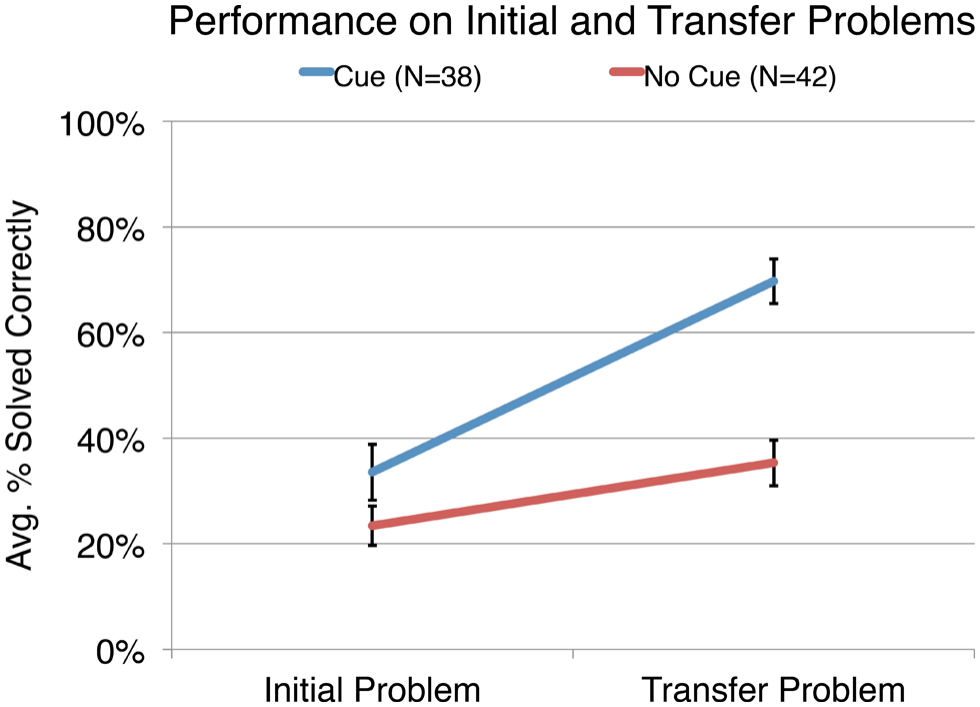
**The average percentage of initial and transfer problems answered correctly by participants in the cued and uncued conditions.** The error bars indicate ± 1 SE of the mean.

We were first interested in the pedagogical effectiveness of the visual cues in helping participants correctly solve and reason about the problems. **Figure 3** shows the average percentage of initial and transfer problems solved correctly (correct in terms of both the answer and explanation) by the participants in the cued and uncued conditions. On average, participants in the uncued condition correctly solved 23.4% of initial problems and 35.3% of transfer problems. Participants in the cued condition correctly solved an average of 33.6% of initial problems and 69.7% of transfer problems. To compare the performance of the cued and uncued participants, a repeated measures ANOVA was conducted with the proportion of the initial and transfer problems correctly solved as the within-subjects factor and the condition as the between-subjects factor.

The results of the ANOVA indicated that there was a main effect of problem, *F*(1,78) = 64.55, *p* < 0.001 and of condition, *F*(1,78) = 16.45, *p* < 0.001. These main effects were qualified by a significant interaction, *F*(1,78) = 16.45, *p* < 0.001 indicating that participants in the cued and uncued conditions performed differently depending on the problem. Probing the interaction we find that there was no significant difference in the average proportion of initial problems answered correctly by participants in the cued and uncued conditions, *F*(1,78) = 3.42, *p* = 0.068. However, those in the cued condition, on average, correctly solved a significantly larger proportion of transfer problems than those in the uncued condition, *F*(1,78) = 39.38, *p* < 0.001, *d* = 1.07. Both those in the cued and uncued conditions showed a significant increase from initial to transfer, *F*(1,78) = 69.11, *p* < 0.001, *d* = 1.23 and *F*(1,78) = 8.28, *p* = 0.005, *d* = 0.45, respectively. After watching cues on the training problems, participants in the cued condition solved nearly twice the proportion of transfer problems correctly as compared to participants in the uncued condition. These results demonstrate that the visual cues significantly improve performance on the transfer problem. More importantly, the results suggest that the visual cues promote higher level cognition as evinced by the improved performance on the transfer problem.

### COMPARING THE ATTENTION OF CORRECT AND INCORRECT SOLVERS ON THE INITIAL PROBLEM

**FIGURE 4:**
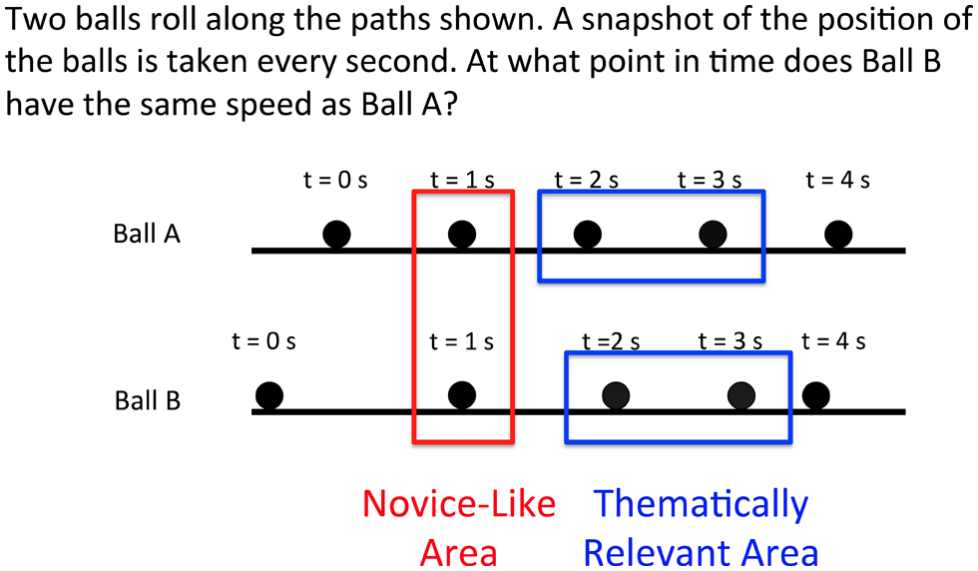
**An example of the thematically relevant area and novice-like area in an initial problem.** Respectively, these areas are associated with the correct response (time interval when the balls travel the same distance) and most common incorrect response (time when the balls are at the same position).

Madsen et al. (2012) showed that correct and incorrect solvers differ in their allocation of visual attention while solving problems with diagrammatic features consistent with novice-like answers in addition to thematically relevant regions. Specifically, participants who answer the problems correctly spend significantly more time attending to the thematically relevant areas and a significantly smaller proportion of time attending to the features associated with the novice-like answers than participants who answer the problems incorrectly. The novice-like and thematically relevant areas in the problems investigated in this study are depicted in **Figure 4**. We performed a similar analysis to determine if the correctness on the initial problem could be attributed to participants’ attention in the thematically relevant and novice-like regions.

To analyze the eye movements, areas of interest (AOI) were drawn around the thematically relevant and novice-like areas associated with each problem with a border of 1.1° of visual angle. The size of the areas was determined by using an error propagation technique (Preston and Dietz, 1991) which took into account both the eye tracker’s accuracy and the spatial extent of the central fovea (0.5° and 1° of visual angle, respectively). When comparing eye movements across several problems, the physical sizes of the thematically relevant and novice-like areas are non-constant and should be normalized. To do this, we divided the percentage of dwell time in the AOI by the percentage of screen that the AOI subtends. This produced a new measure, the percentage of total dwell time divided by the percentage of total area, which is described as the domain relative ratio (Fletcher-Watson et al., 2008).

**FIGURE 5:**
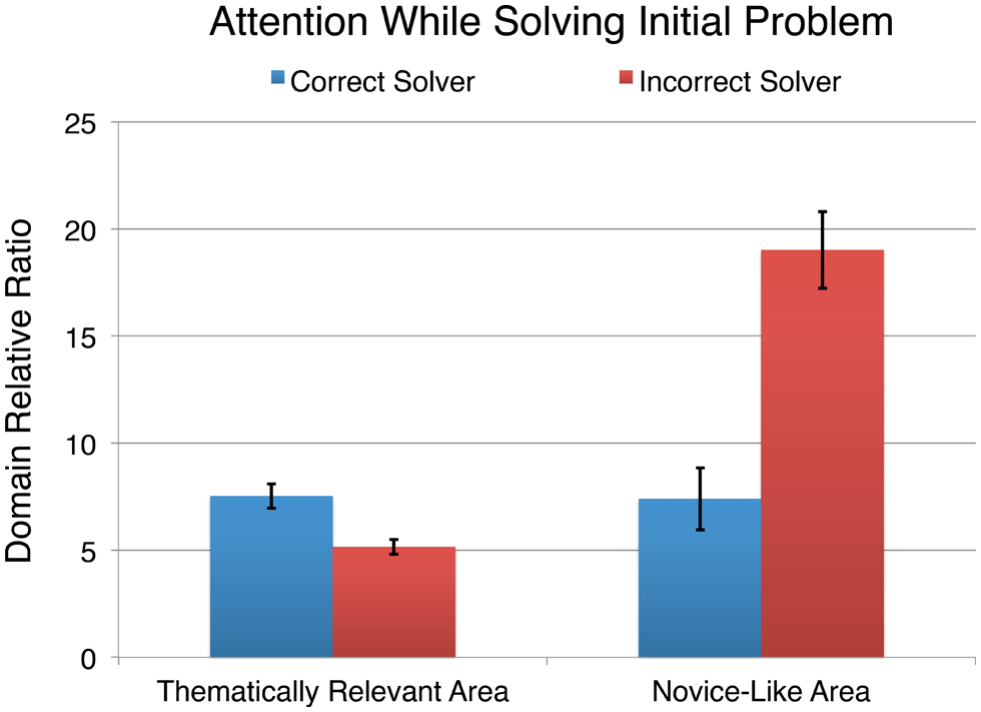
**The domain relative ratio (percentage of dwell time divided by the percentage of area the AOI encompasses) in the thematically relevant and novice-like areas on the initial problem by the correctness of response.** Error bars indicate ± 1 standard error of the mean.

**Figure 5** shows the domain relative ratio spent by correct and incorrect solvers in the thematically relevant and novice-like areas while they solved the initial problem in each set. To compare the proportion of time that correct and incorrect solvers spent attending to the thematically relevant and novice-like areas, we conducted two one-way ANOVAs with the domain relative ratio as the dependent measure and correctness as the between-subjects factor. The results indicate that those who solved the initial problem correctly had a significantly larger domain relative ratio in the thematically relevant area, *F*(1,318) = 13.20, *p* < 0.001, *d* = 0.44 while simultaneously spending a significantly smaller domain relative ratio in the novice-like area, *F*(1,318) = 14.85, *p* < 0.001, *d* = 0.47. These results are consistent with Madsen et al’s. (2012) findings.

### ATTENTION WHILE THE CUE PLAYED

Participants in the cued condition were required to play the cues on the training problems at least once, but were allowed to replay the cue as many times as desired. The vast majority of the time the participants chose to play the cues just once, accounting for 90.4% of all training problems solved. The cue was played twice 8.1% of the time, 55.4% of which occurred during the first training problem in a set.

We investigated whether participants who most needed to see the cue (namely those who provided an incorrect response to the immediately preceding problem in the set) actually watched the cue while it was on screen. We found that those who switched to a correct response had, on average, a domain relative ratio of 16.5 spent watching the cue while it was on screen, while those who retained an incorrect response had a domain relative ratio of 13.2. To compare these values, a one-way ANOVA was conducted with the domain relative ratio as the dependent measure and correctness pattern as the between-subjects factor. The results indicated that the cued participants who switched to a correct response spent a significantly larger proportion of time per area watching the cue, *F*(1,277) = 7.71, *p* = 0.006, *d* = 0.34. This result demonstrates that watching the cue more closely can be tied to participants switching from an incorrect to correct response.

### CHANGES IN EYE MOVEMENTS AMONG PARTICIPANTS WHO DEMONSTRATED LEARNING

Thus far, we have demonstrated that cues can be an effective learning tool and that there is a link between the correctness of a student’s response and their allocation of attention while solving the problem. We now consider the subset of participants who we can reasonably assume learned something—that is, those who answered the initial problem incorrectly, but after working through the training problems were successful in correctly solving the transfer problem. Each case in which a participant demonstrated learning was treated as an independent observation in the analyses described later in this section. Across all problem sets, we have 66 cases (34 unique participants) of this occurring in the cued group and 30 cases (21 unique participants) in the uncued group, corresponding to 89.5% and 50.0% of participants in the cued and uncued groups, respectively. There was significantly greater number of participants in the cued condition following this pattern than in the uncued condition, χ^2^(1, *N* = 320) = 24.83, *p* < 0.001, *V* = 0.279. The number of participants demonstrating learning on one or more problems is provided in **Table 1**.

**Table 1:** The number of problem sets in which participants demonstrated learning in the cued and uncued conditions.

Condition	Number of participants demonstrating learning on			
	1 Problem set	2 Problem sets	3 Problem sets	4 Problem sets
Cue (*N* = 38)	11	16	5	2
No cue (*N* = 42)	13	7	1	0

As indicated by **Table 1**, the analyses reported below contained cases in which some participants contributed only a single observation, whereas other participants contributed multiple observations, across all problem sets. Thus, we did not include “problem set” as a within-subjects factor in our analyses due to missing data. Because having different numbers of observations across problem sets as a function of participants could create additional within-subject dependencies in our analyses, we carried out a robustness check. Specifically, we carried out the analyses discussed in this section on a randomly selected subsample of the data in which no participant contributed more than a single observation. The results of these additional analyses showed the same pattern of results reported below—all significant main effects and interactions reported below were also significant with only the randomly chosen subsample. Therefore, for all analyses reported in this section, we have included the full data set shown in **Table 1**.

#### Attention in the thematically relevant area

**FIGURE 6:**
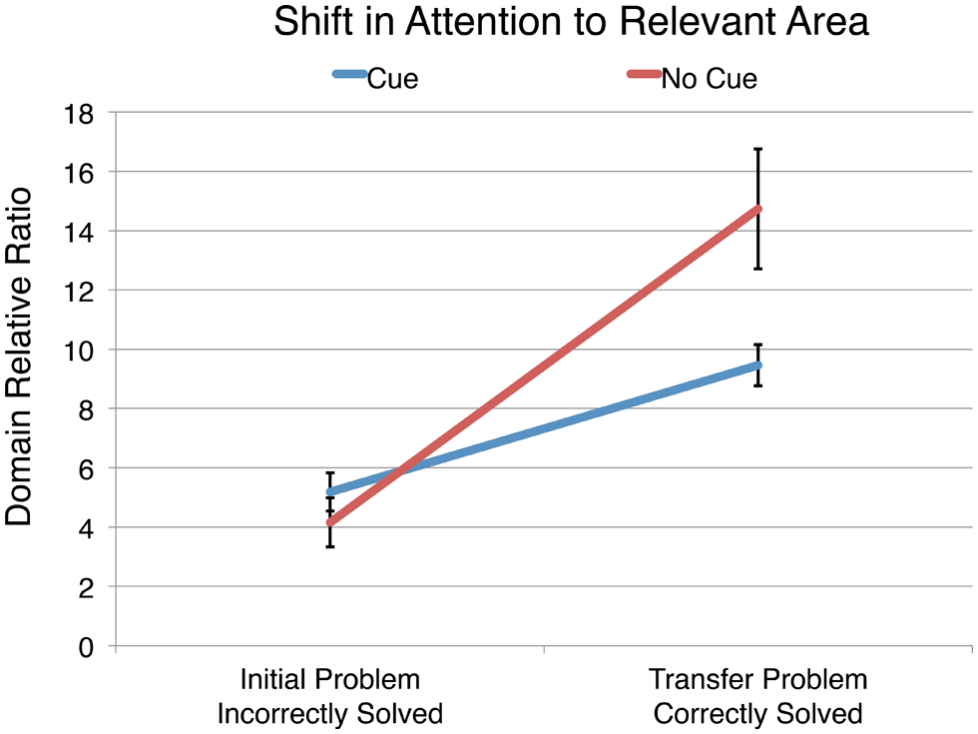
**The domain relative ratio (percentage of dwell time divided by the percentage of area the AOI encompasses) in the thematically relevant area on the initial and transfer problems for those who improved from the initial to transfer problem.** The error bars indicate ± 1 SE of the mean.

After finding that correct solvers spent a significantly larger proportion of their time attending to the relevant area, we wanted to see if the participants who demonstrated learning had an increased domain relative ratio in the transfer problem. **Figure 6** shows the domain relative ratio that cued and uncued participants spent in the relevant area on the initial and transfer problems.

A repeated measures ANOVA with domain relative ratio in the thematically relevant area as the dependent measure and condition as the between-subjects factor was conducted. There was a significant increase in the domain relative ratio in the relevant area from the initial to the transfer problem, *F*(1,94) = 56.41, *p* < 0.001 and a significant main effect of condition, *F*(1,94) = 4.12, *p* = 0.045. However, these main effects are qualified by a significant interaction, *F*(1,94) = 10.17, *p* = 0.002, indicating that the cued and uncued groups performed differently depending on the problem. Probing the interaction we find that the domain relative ratio of both the cued and uncued groups increased significantly from initial to transfer problem, *F*(1,94) = 14.94, *p* < 0.001, *d* = 0.79 and *F*(1,94) = 41.63, *p* < 0.001, *d* = 1.35, respectively. However, while there was no significant difference between the cued and uncued conditions on the initial problem, *F*(1,94) < 1, the uncued condition had a significantly higher domain relative ratio in the relevant area than the cued condition on the transfer problem, *F*(1,94) = 14.25, *p* < 0.001, *d* = 0.65.

Inconsistent with the *processing prioritization* hypothesis, among participants who showed evidence of learning (i.e., improved performance on the transfer problem relative to the initial problem), those who saw cues had a significantly smaller domain relative ratio in the relevant area on the transfer problem than those who did not see cues. This is despite the fact that solvers in the cued condition received training to attend the relevant area. This result is surprising, and seems to pose a paradox. Namely, why would those trained to attend to the relevant area spend less time attending to the relevant area than those who were not trained to do so? A possible solution of this paradox is given by the *automatization* hypothesis, namely that those who were given training with the cues may have developed greater automaticity in extracting the relevant information, and thus spent proportionally less time attending to the relevant area of the transfer problem than those solvers who did not receive the cued training (i.e., the uncued participants).

#### Automaticity in extracting relevant information

**FIGURE 7:**
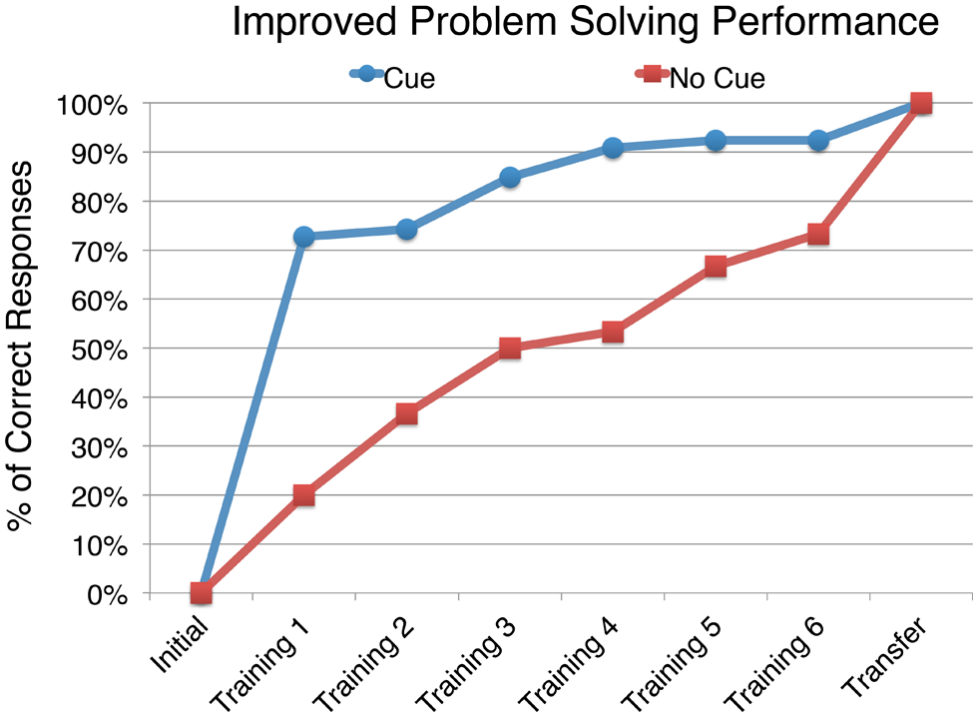
**The performance on each problem for the subset of participants who shifted from an incorrect response on the initial problem to a correct response on the transfer problem**.

We hypothesized that the reason the cued group had a smaller domain relative ratio in the thematically relevant area on the transfer problem than the uncued group was because the cued group was able to more easily extract the relevant information from the diagram, namely the automatization hypothesis. If so, evidence for the increased efficiency of relevant information extraction should be found by examining their performance on the training problems. Specifically, participants in the cued condition should have had greater success in extracting the relevant information over more trials than participants in the uncued condition, which would then produce greater automaticity of extracting relevant information for the cued group. A test of this hypothesis is shown in **Figure 7**, which shows student performance across all problems within the sets.

Consistent with the automatization hypothesis, among cued participants who answered the initial problem incorrectly, we find that 73% were able to correctly solve the first training problem, and the proportion increased to 92% by the sixth training problem. In contrast, only 20% of the uncued group answered the first training problem correctly, and by the sixth problem 73% were correct. Because a larger proportion of participants in the cued group were able to answer the training problems correctly, they had more practice doing so, and thus gained more automaticity in extracting the relevant information. In addition, the increase in percentage of correct responses in the two groups from the sixth training problem to the transfer problem was greater for the uncued group – that is, getting the transfer problem correct was a bigger leap for more of those in the uncued condition than those in the cued condition.

To statistically compare the cued and uncued participants’ performance depicted in **Figure 7**, a survival analysis was conducted. To do this, the training problem number in which the participant switched to providing only correct responses was considered. Comparing the resulting survival curves using a log-rank test indicates that the participants who saw cues on the training problems switched to a correct response significantly earlier than those in the uncued group. χ^2^(1, *N* = 96) = 16.17, *p* < 0.001. Altogether, these conditions likely led to the cued group having greater ease of extracting the relevant information (indicated by the smaller domain relative ratio) on the transfer problem than the uncued group (as shown in **Figure 6**).

#### Average fixation duration in the thematically relevant area

A further test of the automatization hypothesis is in terms of the successful problem solvers’ average fixation durations. We would expect that increased ease of extracting the relevant information, namely greater automaticity, would be associated with shorter fixation durations in the relevant area. **Table 2** shows the average fixation durations of the cued and uncued participants in the relevant area and entire diagram for the transfer problems. (Note that there was no cueing on the transfer problem, even in the cued condition.)

**Table 2:** The average fixation durations (in ms) ± 1 SE of the mean for the cued and uncued groups in the relevant area and entire diagram while viewing the transfer problems.

Area of interest	Avg. fixation duration in ms (mean ± SE)	
	Cued *(N* = 66)	Uncued *(N* = 30)
Relevant area	239 ± 10	280 ± 15
Entire diagram	227 ± 6	219 ± 7

The average fixation durations of participants while solving the transfer problems were compared using a 2 (cue vs. no cue) × 2 (entire diagram vs. relevant area) ANOVA. The results are summarized in **Table 3**.

**Table 3:** Results of a 2 (cue vs. no cue) × 2 (entire diagram vs. relevant area) ANOVA comparing the average fixation duration on the transfer problem.

Effect	*F* (1,94)	*p*	*d*
Main effect of area (entire diagram vs. relevant area)	23.33	<0.001	0.42
Main effect of condition (cued vs. uncued)	1.72	0.193	–
Interaction of area × condition	10.87	0.001	–
Simple effect of area (cue only)	1.88	0.174	–
Simple effect of area (uncued only)	24.01	<0.001	0.96
Simple effect of condition (entire diagram only)	<1	n.s.	–
Simple effect of condition (relevant area only)	10.90	0.001	0.64

A significant interaction between the condition and area of interest was found. Probing the significant interaction, we find that within the relevant area, cued participants had significantly shorter mean fixation durations than those in the uncued condition. This is consistent with the hypothesis that the cued participants had indeed developed greater automaticity in extracting information from the relevant area than the uncued participants. We also found that uncued participants had significantly larger fixation durations in the relevant area of the diagram compared to their average fixation durations when considering the entire diagram. However, for those in the cued group, the average fixation duration in the relevant area is not distinguishable from the rest of the problem. The combination of these results indicates that cued participants experience a greater ease of extraction of the relevant information on the transfer problem, as evidenced by their lower fixation durations. This would explain why the cued group spends a smaller proportion of time attending to the relevant area on the transfer problem (as shown in **Figure 6**).

## DISCUSSION

In this study we investigated the relationship between the low-level perceptual processes involved in overt attentional selection by visual cues, on the one hand, and the high-level cognitive processes involved in solving physics problems. Eye movements can be used to elucidate *what* information within a diagram is being processed and *when* that information is being processed. This allows for us to investigate how participants’ attention changes over time and relevant cognitive processes associated with problem solving. In the following sections, we revisit our hypotheses and discuss our findings.

### CORRECTNESS

Based on the changes we made in the current study in comparison to our previous studies, we were able to show that visual cues did indeed improve problem solving on the transfer problems. The changes we made were those suggested by consideration of both Ohlsson’s (1992) representational change theory and de Koning and colleagues framework of attention cueing (de Koning et al., 2009). In particular, we told solvers that the cues were meant to help them, we provided correctness feedback to induce impasses among those who originally had an incorrect solution path, and we included visual cues that facilitated not only attentional selection of relevant information, but also integration of that information across different regions of the problem. Doing so indeed facilitated solver’s ability to re-represent the problem in a meaningful way allowing for the extraction of the relevant information and thus improved performance.

We found a significantly greater proportion of participants who received training with visual cues were able to subsequently correctly solve the transfer problem without cues than those who received training in the uncued condition. We observed that both the cued and uncued groups performed similarly on the initial problem and both experienced significant increase in performance from the initial to transfer problem. However, nearly twice as many participants in the cued condition were able to correctly solve the transfer problem as compared to participants in the uncued condition (69.7 vs. 35.5%, respectively). This amounted to more than one standard deviation difference between the groups. These results provide evidence that the visual cues facilitated the participants to re-represent the problem enabling them to break an impasse and solve the problem correctly. More importantly, these results provide evidence, consistent with previous studies (Thomas and Lleras, 2009) that manipulation of low-level eye movements can influence high level cognition involved in problem solving. Nevertheless, there is a critically important difference between the results of our studies and those of Grant and Spivey (2003) and Thomas and Lleras (2007, 2009), who proposed the provocative idea that simply having the viewer’s low-level attentional movements embody a problem’s solution is sufficient to facilitate finding the correct solution. Specifically, our research, including both the current and previous studies (Madsen et al., 2013a,b) has shown that while attending to relevant information in a problem is a necessary condition for correctly solving the problem, it is generally not sufficient to correctly solving it. The current study has specifically shown that cues, which both draw attention to solution-relevant information, and facilitate organizing and integrating it, facilitate both immediate problem solving and generalization of that ability to new problems. In addition, the current study shows that when such cues are used across multiple problems, solvers can automatize the extraction of problem-relevant information.

### CHANGES IN EYE MOVEMENTS

In the current study, we were particularly interested in the online processes linking overt attentional selection with higher-level cognitive processes involved in problem solving. Thus, we explored how participants’ attention in the relevant area of the diagram changed from the initial problem to the transfer problem. For this set of analyses, we considered the subgroup of participants who demonstrated improvement in their problem solving from the initial to transfer problem. We focused on this subgroup as they were the ones who through the improvement of their responses from the initial to transfer problem, showed evidence that higher order cognitive processes were online.

We presented two competing hypotheses for how cued and uncued participants’ attention in the thematically relevant area of the diagram would compare on the transfer problem. The *processing priority hypothesis* was that through training of attentional prioritization, solvers in the cued condition would spend a *larger* percentage of dwell time per percentage of area attending to the relevant features on the transfer problem, namely a *higher* domain relative ratio in the relevant area of the transfer problem for the cued group compared to the uncued group. Alternatively, the *automatization hypothesis* was that repeated training in attending to and extracting relevant information from a problem type would increase participants’ efficiency in doing so, and therefore participants in the cued condition would have shorter fixation durations on the relevant features on the transfer problem than those in the uncued group.

We found that successful problem solvers attend to the relevant information in the diagram significantly more than unsuccessful solvers. When provided with cues on the training problems, participants who successfully switch to correct responses overtly attend to the cue significantly more closely. Among the subset of participants who improved their performance from the initial to transfer problem, we found that the cued group nearly doubled their percentage of dwell time per percentage of area in the thematically relevant area while those in the uncued condition more than tripled the domain relative ratio in the relevant area.

While the cued participants had a significantly larger domain relative ratio in the relevant area of the transfer problem than they did while solving the initial problem, it was still significantly less than the domain relative ratio of uncued group on the transfer problem. To investigate if this result could be tied to the cued group having developed an increased ease of extraction of the relevant information, we examined the participants’ performance on the training problems as well as their average fixation durations while solving the initial and transfer problems.

In examining the training problem performance of those who improved from the initial to transfer problem, we found that the cued group showed a significant increase on the first training problem, followed by a more gradual increase on subsequent training problems. By contrast the uncued group showed a slower increase from the first training problem through the sixth training problem with nearly the same proportion of successful solvers on the sixth training problem that the cued group had on the first. This difference in the trajectories of the cued and uncued subgroups going from incorrectly solving the initial problem to correctly solving the transfer problem indicates that participants in the cued group had acquired greater practice than those in the uncued group in extracting information from the relevant area because they correctly solved a larger proportion of training problems. Therefore, the cued group would have achieved greater automaticity in extracting the relevant problem information than the uncued group. This conclusion is consistent with our finding that the cued group showed a lower mean fixation duration in the relevant area on the transfer problem compared to the uncued group.

An open question for further research is the degree to which the cueing effects in the current study were predicated on telling the solvers that the cues were helpful. Based on the previous results of Thomas and Lleras (2007, 2009), in our previous studies we did not inform solvers that the cues would be helpful, but we found only moderate effects of visual cueing on overt attention and successful problem solving. The current study did tell solvers that the cues were “hints” meant to help them, and found strong effects of visual cueing on both overt attention and successful problem solving. Further research can experimentally vary whether solvers are told about the helpfulness of cues and see the degree to which this is important.

A further open question is the degree to which forcing the initially incorrect solvers into an impasse, either explicitly by providing them with correctness feedback, or implicitly by providing them with visual cues that focus on information they have previously ignored, is critical for creating strong effects of cueing on attentional selection and successful insight problem solving. The current study found that both cueing and correctness feedback facilitated solvers to make the transition from incorrect solution paths to correct solution paths. Interestingly, cueing by itself was more effective than feedback by itself. This raises the question of whether both created impasses. Further research will be needed to create on-line measures of impasse in both cueing and feedback conditions to determine the effects of each on entering an impasse during insight problem solving.

In summary, the current study has shown two important findings. First, short duration visual cues can improve problem solving performance on a variety of insight physics problems, including transfer problems that do not share the surface features of the training problems, but do share the underlying solution path. In other words, visual cues can facilitate solvers to re-represent a problem and overcome impasse thereby enabling them to correctly solve a problem. These cueing effects on problem solving were not predicated upon the solvers’ overt or covert attentional shifts necessarily embodying the solution to the problem. Instead, the cueing effects were predicated upon having solvers attend to and integrate relevant information in the problems into a solution path. Second, these short duration visual cues when administered repeatedly over multiple training problems resulted in participants becoming more efficient at extracting the relevant information on the transfer problem, showing that such cues can improve the automaticity with which solvers extract relevant information from a problem. These results, when combined with those of our previous studies (Madsen et al., 2013a,b) suggest that low-level attentional selection processes provide a necessary gateway for relevant information to be used in problem solving, but are generally not sufficient for correct problem solving. Instead, factors that lead a solver to an impasse (e.g., correctness feedback) and to organize and integrate problem information (e.g., organization and integration cues) also greatly facilitate arriving at correct solutions. We are currently studying the specific effects of these factors on problem solving within the context of a model of the role of visual cueing in conceptual problem solving (Rouinfar et al., 2014). Further research along these lines will enable us to more precisely understand the role of lower-level attentional selection in higher-level problem solving.

## Conflict of Interest Statement

The authors declare that the research was conducted in the absence of any commercial or financial relationships that could be construed as a potential conflict of interest.

## References

[B1] Awh E. Jonides J. Reuter-Lorenz P. A. (1998). Rehearsal in spatial working memory. *J. Exp. Psychol. Hum. Percept.* *Perform.* 24 780–790 10.1037/0096-1523.24.3.780 9627416

[B2] Baddeley A. (1994). The magical number seven: still magic after all these years? *Psychol. Rev.* 101 353–356 10.1037/0033-295X.101.2.353 8022967

[B3] Baluch F. Itti L. (2011). Mechanisms of top-down attention. *Trends Neurosci.* 34 210–224 10.1016/j.tins.2011.02.003 21439656

[B4] Belopolsky A. V. Kramer A. F. Godijn R. (2008). Transfer of information into working memory during attentional capture. *Vis. Cogn.* 16 409–418 10.1080/13506280701695454

[B5] Bilalić M. McLeod P. Gobet F. (2008). Why good thoughts block better ones: the mechanism of the pernicious Einstellung (set) effect. *Cognition* 108 652–661 10.1016/j.cognition.2008.05.005 18565505

[B6] Boehnke S. E. Munoz D. P. (2008). On the importance of the transient visual response in the superior colliculus. *Curr. Opin. Neurobiol.* 18 544–551 10.1016/j.conb.2008.11.004 19059772

[B7] Borchers H.-W. Ewert J.-P. (1979). Correlation between behavioral and neuronal activities of toads *Bufo bufo* (L.) in response to moving configurational prey stimuli. *Behav. Process.* 4 99–106 10.1016/0376-6357(79)90026-3 24924977

[B8] Cameron E. L. Tai J. C. Carrasco M. (2002). Covert attention affects the psychometric function of contrast sensitivity. *Vision Res.* 42 949–967 10.1016/S0042-6989(02)00039-1 11934448

[B9] Carmi R. Itti L. (2006). Visual causes versus correlates of attentional selection in dynamic scenes. *Vision Res.* 46 4333–4345 10.1016/j.visres.2006.08.019 17052740

[B10] Carrasco M. Penpeci-Talgar C. Eckstein M. (2000). Spatial covert attention increases contrast sensitivity across the CSF: support for signal enhancement. *Vision Res.* 40 1203–1215 10.1016/S0042-6989(00)00024-9 10788636 PMC3825514

[B11] Carrasco M. Williams P. E. Yeshurun Y. (2002). Covert attention increases spatial resolution with or without masks: support for signal enhancement. *J. Vis.* 2 467–479 10.1167/2.6.4 12678645

[B12] Caspi A. Beutter B. R. Eckstein M. P. (2004). The time course of visual information accrual guiding eye movement decisions. *Proc. Natl. Acad. Sci. U.S.A.* 101 13086–13090 10.1073/pnas.0305329101 15326284 PMC516521

[B13] Charness N. Reingold E. M. Pomplun M. Stampe D. M. (2001). The perceptual aspect of skilled performance in chess: evidence from eye movements. *Mem. Cogn.* 29 1146–1152 10.3758/BF03206384 11913751

[B14] Cowan N. (2001). The magical number 4 in short-term memory: a reconsideration of mental storage capacity. *Behav. Brain Sci.* 24 87–185 10.1017/S0140525X01003922 11515286

[B15] D’Esposito M. Postle B. R. Ballard D. Lease J. (1999). Maintenance versus manipulation of information held in working memory: an event-related fMRI study. *Brain Cogn.* 41 66–86 10.1006/brcg.1999.1096 10536086

[B16] de Koning B. B. Tabbers H. K. Rikers R. M. J. P. Paas F. (2009). Towards a framework for attention cueing in instructional animations: guidelines for research and design. *Educ. Psychol. Rev.* 21 113–140 10.1007/s10648-009-9098-7

[B17] Deubel H. Schneider W. X. (1996). Saccade target selection and object recognition: evidence for a common attentional mechanism. *Vision Res.* 36 1827–1837 10.1016/0042-6989(95)00294-4 8759451

[B18] Duncker K. (1945). On problem-solving processes. *Psychol. Monogr.* 58 i–113. 10.1037/h0093599

[B19] Einhäuser W. Rutishauser U. Koch C. (2008). Task-demands can immediately reverse the effects of sensory-driven saliency in complex visual stimuli. *J. Vis.* 8 1–19 10.1167/8.2.2 18318628

[B20] Eivazi S. Bednarik R. (2010). “Inferring problem solving strategies using eye-tracking: system description and evaluation,” in *Proceedings of the 10th Koli Calling International Conference on Computing Education Research* 55–61 10.1145/1930464.1930472

[B21] Eivazi S. Bednarik R. (2011). “Predicting problem-solving behavior and performance levels from visual attention data,” in *Proceedings of the 2^nd^ Workshop on Eye Gaze in Intelligent Human Machine Interaction at IUI 2011* 9–16

[B22] Epelboim J. Suppes P. (2001). A model of eye movements and visual working memory during problem solving in geometry. *Vision Res.* 41 1561–1574 10.1016/S0042-6989(00)00256-X 11343722

[B23] Everling S. Fischer B. (1998). The antisaccade: a review of basic research and clinical studies. *Neuropsychologia* 36 885–899 10.1016/S0028-3932(98)00020-7 9740362

[B24] Findlay J. Walker R. (1999). A model of saccade generation based on parallel processing and competitive inhibition. *Behav. Brain Sci.* 22 661–721 10.1017/S0140525X99002150 11301526

[B25] Fischer J. Whitney D. (2009). Attention narrows position tuning of population responses in V1. *Curr. Biol.* 19 1356–1361 10.1016/j.cub.2009.06.059 19631540 PMC2757109

[B26] Fletcher-Watson S. Findlay J. M. Leekam S. R. Benson V. (2008). Rapid detection of person information in a naturalistic scene. *Perception* 37 571–583 10.1068/p5705 18546664

[B27] Foulsham T. Underwood G. (2007). How does the purpose of inspection influence the potency of visual salience in scene perception? *Perception* 36 1123–1138 10.1068/p5659 17972478

[B28] Glaholt M. G. Rayner K. Reingold E. M. (2012). The mask-onset delay paradigm and the availability of central and peripheral visual information during scene viewing. *J. Vis.* 12 1–19 10.1167/12.1.9 22247221

[B29] Grant E. R. Spivey M. J. (2003). Eye movements and problem solving: guiding attention guides thought. *Psychol. Sci.* 14 462–466 10.1111/1467-9280.02454 12930477

[B30] Guitton D. Buchtel H. A. Douglas R. M. (1985). Frontal lobe lesions in man cause difficulties in suppressing reflexive glances and in generating goal directed saccades. *Exp. Brain Res.* 58 455–472 10.1007/BF00235863 4007089

[B31] Hale S. Myerson J. Rhee S. H. Weiss C. S. Abrams R. A. (1996). Selective interference with the maintenance of location information in working memory. *Neuropsychology* 10 228–240 10.1037/0894-4105.10.2.228

[B32] Henderson J. M. (1992). “Visual attention and eye movement control during reading and picture viewing,” in *Eye Movements and Visual Cognition: Scene Perception and Reading* ed. Rayner K. (New York: Springer) 260–283

[B33] Henderson J. M. (1993). “Visual attention and saccadic eye movements,” in *Perception and Cognition: Advances in Eye Movement Research. Studies in Visual Information Processing* ed. Gery d’Ydewalle J. V. R. (Amsterdam: North-Holland/Elsevier Science Publishersx) 37–50

[B34] Henderson J. M. Brockmole J. R. Castelhano M. S. Mack M. L. (2007). “Visual saliency does not account for eye movements during visual search in real-world scenes,” in *Eye Movements: A Window on Mind and Brain* ed. Gompel R. V. Fischer M. Murray W. Hill R. W. (Amsterdam: Elsevier) 537–562

[B35] Hollingworth A. Henderson J. M. (2002). Accurate visual memory for previously attended objects in natural scenes. *J. Exp. Psychol. Hum. Percept. Perform.* 28 113–136 10.1037//0096-1523.28.1.113

[B36] Irwin D. E. Colcombe A. M. Kramer A. F. Hahn S. (2000). Attentional and oculomotor capture by onset, luminance, and color singletons. *Vision Res.* 40 1443–1458 10.1016/S0042-6989(00)00030-4 10788651

[B37] Itti L. Koch C. (2000). A saliency-based search mechanism for overt and covert shifts of visual attention. *Vision Res.* 40 1489–1506 10.1016/S0042-6989(99)00163-7 10788654

[B38] Jones G. (2003). Testing two cognitive theories of insight. *J. Exp. Psychol. Learn. Mem. Cogn.* 29 1017–1027 10.1037/0278-7393.29.5.1017 14516232

[B39] Knoblich G. Ohlsson S. Raney G. E. (2001). An eye movement study of insight problem solving. *Mem. Cogn.* 29 1000–1009 10.3758/BF03195762 11820744

[B40] Knoblich G. Öllinger M. Spivey M. (2005). “Tracking the eyes to obtain insight into insight problem solving,” in *Cognitive Processes in Eye Guidance* ed. Underwood G. (Oxford: Oxford University Press) 355–375

[B41] Kowler E. Anderson E. Dosher B. Blaser E. (1995). The role of attention in the programming of saccades. *Vision Res.* 35 1897–1916 10.1016/0042-6989(94)00279-U 7660596

[B42] Kustov A. A. Robinson D. L. (1996). Shared neural control of attentional shifts and eye movements. *Nature* 384 74–77 10.1038/384074a0 8900281

[B43] Larson A. M. Freeman T. E. Ringer R. V. Loschky L. C. (2014). The spatiotemporal dynamics of scene gist recognition. *J. Exp. Psychol. Hum. Percept. Perform.* 40 471–487 10.1037/a0034986 24245502

[B44] Lin J. H. Lin S. J. (2014). Tracking eye movements when solving geometry problems with handwriting devices. *J. Eye Mov. Res.* 7 1–15

[B45] Luck S. J. Vogel E. K. (1997). The capacity of visual working memory for features and conjunctions. *Nature* 390 279–281 10.1038/36846 9384378

[B46] Madsen A. M. Larson A. M. Loschky L. C. Rebello N. S. (2012). Differences in visual attention between those who correctly and incorrectly answer physics problems. *Phys. Rev. ST Phys. Educ. Res.* 8:010122-13 10.1103/PhysRevSTPER.8.010122

[B47] Madsen A. M. Rouinfar A. Larson A. M. Loschky L. C. Rebello N. S. (2013a). Can short duration visual cues influence students’ reasoning and eye movements in physics problems? *Phys. Rev. ST Phys. Educ. Res.* 9 020104–020116 10.1103/PhysRevSTPER.9.020104

[B48] Madsen A. M. Rouinfar A. Larson A. M. Loschky L. C. Rebello N. S. (2013b). Do perceptually salient elements in physics problems influence students’ eye movements and answer choices? *Paper presented at the American Institute of Physics Conference Series* Philadelphia, PA

[B49] Maier N. R. F. (1931). Reasoning in humans. II. The solution of a problem and its appearance in consciousness. *J. Comp. Psychol.* 12 181–192 10.1037/h0071361

[B50] Mital P. Smith T. J. Hill R. Henderson J. M. (2010). Clustering of gaze during dynamic scene viewing is predicted by motion. *Cognit. Comput.* 3 5–24 10.1007/s12559-010-9074-z

[B51] Mitchell J. P. Macrae C. N. Gilchrist I. D. (2002). Working memory and the suppression of reflexive saccades. *J. Cogn. Neurosci.* 14 95–103 10.1162/089892902317205357 11798390

[B52] Nuthmann A. Smith T. J. Engbert R. Henderson J. M. (2010). CRISP: a computational model of fixation durations in scene viewing. *Psychol. Rev.* 117 382–405 10.1037/a0018924 20438231

[B53] Ohlsson S. (1992). “Information-processing explanations of insight and related phenomena,” in *Advances in the Psychology of Thinking* eds Keane T. Gilhooly K. J. (London: Harvester-Wheatsheaf) 1–44

[B54] Pertzov Y. Avidan G. Zohary E. (2009). Accumulation of visual information across multiple fixations. *J. Vis.* 9 2–12 10.1167/9.10.2 19810783

[B55] Pestilli F. Carrasco M. Heeger D. J. Gardner J. L. (2011). Attentional enhancement via selection and pooling of early sensory responses in human visual cortex. *Neuron* 72 832–846 10.1016/j.neuron.2011.09.025 22153378 PMC3264681

[B56] Preston D. W. Dietz E. R. (1991). *The Art of Experimental Physics.* New York: John Wiley & Sons

[B57] Rayner K. (1998). Eye movements in reading and information processing: 20 years of research. *Psychol. Bull.* 124 372–422 10.1037/0033-2909.124.3.372 9849112

[B58] Reed S. K. (1993). “A schema-based theory of transfer,” in *Transfer on Trial: Intelligence, Cognition and Instruction* eds Detterman D. K. Sternberg R. J. (Norwood, NJ: Abex) 39–67

[B59] Reingold E. M. Charness N. Pomplun M. Stampe D. M. (2001). Visual span in expert chess players: evidence from eye movements. *Psychol. Sci.* 12 48–55 10.1111/1467-9280.00309 11294228

[B60] Rouinfar A. Agra E. Larson A. M. Loschky L. C. Rebello N. S. (2014). Can visual cues and correctness feedback influence students’ reasoning? *Paper presented at the American Institute of Physics Conference Series*.

[B61] Schneider W. Shiffrin R. M. (1977). Controlled and automatic human information processing: I. Detection, search, and attention. *Psychol. Rev.* 84 1–66 10.1037/0033-295X.84.1.1

[B62] Shinoda H. Hayhoe M. M. Shrivastava A. (2001). What controls attention in natural environments? *Vision Res.* 41 3535–3545 10.1016/S0042-6989(01)00199-7 11718793

[B63] Susac A. Bubic A. Kaponja J. Planinic M. Palmovic M. (2014). Eye movements reveal students’ strategies in simple equation solving. *Int. J. Sci. Math. Educ.* 12 555–577 10.1007/s10763-014-9514-4

[B64] Tai R. H. Loehr J. F. Brigham F. J. (2006). An exploration of the use of eye-gaze tracking to study problem solving on standardized science assessments. *Int. J. Res. Methods Educ.* 29 185–208 10.1080/17437270600891614

[B65] Theeuwes J. Godthelp H. (1995). Self-explaining roads. *Saf. Sci.* 19 217–225 10.1016/0925-7535(94)00022-U

[B66] Thomas L. E. Lleras A. (2007). Moving eyes and moving thought: on the spatial compatibility between eye movements and cognition. *Psychon. Bull. Rev.* 14 663–668 10.3758/BF03196818 17972730

[B67] Thomas L. E. Lleras A. (2009). Covert shifts of attention function as an implicit aid to insight. *Cognition* 111 168–174 10.1016/j.cognition.2009.01.005 19249019

[B68] van Diepen P. d’Ydewalle G. (2003). Early peripheral and foveal processing in fixations during scene perception. *Vis. Cogn.* 10 79–100 10.1080/713756668

[B69] Velichkovsky B. M. Rothert A. Kopf M. Dornhöfer S. M. Joos M. (2002). Towards an express-diagnostics for level of processing and hazard perception. *Transp. Res. Part F Traffic Psychol. Behav.* 5 145–156 10.1016/S1369-8478(02)00013-X

[B70] Zelinsky G. J. Loschky L. C. (2005). Eye movements serialize memory for objects in scenes. *Percept. Psychophys.* 67 676–690 16134461 10.3758/bf03193524

[B71] Zelinsky G. J. Loschky L. C. Dickinson C. A. (2011). Do object refixations during scene viewing indicate rehearsal in visual working memory? *Mem. Cogn.* 39 600–613 10.3758/s13421-010-0048-x 21264590

